# Integrative Genetic and Transcriptomic Analysis Reveals Candidate Molecular Differences Among Dementia with Lewy Bodies, Alzheimer’s Disease, and Parkinson’s Disease Dementia

**DOI:** 10.3390/ijms27209173

**Published:** 2026-10-16

**Authors:** Gözde Öztan, Tuğçe İşsever, Halim İşsever

**Affiliations:** 1 Department of Medical Biology, Istanbul Faculty of Medicine, Istanbul University, Topkapı, 34093 Istanbul, Turkey; 2 Turkish Health Institutes Presidency (TUSEB), 34718 Istanbul, Turkey; tugceissever@gmail.com; 3 Department of Public Health, Istanbul Faculty of Medicine, Istanbul University, Topkapı, 34093 Istanbul, Turkey; hissever@istanbul.edu.tr

**Keywords:** dementia with Lewy bodies, Parkinson’s disease dementia, Alzheimer’s disease, transcriptomics, differential gene expression, genetic integration, *SYT11*, *SLC19A2*, *VAMP1*, *COX5B*

## Abstract

Dementia with Lewy bodies (DLB) shares substantial clinical and molecular features with Alzheimer’s disease (AD) and Parkinson’s disease dementia (PDD), complicating the identification of disease-specific molecular alterations. This study aimed to characterize transcriptomic differences among these disorders and to integrate expression findings with published Lewy body dementia (LBD) genetic evidence. Gene-level expression profiles from 42 postmortem prefrontal cortex samples in GSE150696, including controls, AD, DLB, and PDD, were analyzed using age- and sex-adjusted empirical-Bayes models with false discovery rate (FDR) correction, followed by sensitivity analyses, post hoc exploratory candidate selection, and integration with an external LBD multi-omics gene-prioritization dataset. No gene reached FDR < 0.05 in the DLB-versus-AD comparison. In contrast, *SLC19A2*, *VAMP1*, and *COX5B* were lower in DLB than in PDD and were FDR-supported in the primary age- and sex-adjusted model, although their statistical significance was partly model-dependent. A post hoc cross-disease analysis identified 60 exploratory candidates, of which *SYT11* was the only gene overlapping with the published LBD-prioritized gene set. The overall 60-gene set was not significantly enriched for LBD-associated genes. These findings did not demonstrate broad robust disease–disease differences and are consistent with substantial molecular overlap, while remaining compatible with limited statistical power. A limited number of transcriptomic candidates, including the exploratory *SYT11* overlap, warrant independent validation.

## 1. Introduction

Dementia with Lewy bodies (DLB) is a progressive neurodegenerative disorder characterized by dementia accompanied by cognitive fluctuations, recurrent visual hallucinations, rapid eye movement sleep behavior disorder, and spontaneous parkinsonism [1]. Its neuropathological hallmark is the accumulation of misfolded α-synuclein in Lewy bodies and Lewy neurites, frequently accompanied by Alzheimer-type pathology. This clinicopathological complexity remains a major obstacle to accurate diagnosis, molecular stratification, and the development of disease-specific biomarkers.

Parkinson’s disease dementia (PDD) shares extensive clinical and neuropathological features with DLB and, together, the two conditions are commonly considered within the Lewy body dementia spectrum. Their clinical distinction relies mainly on the timing of dementia relative to parkinsonism, with dementia preceding or occurring near motor onset in DLB and developing after established Parkinson’s disease in PDD [1,2]. This operational distinction does not necessarily imply sharply separate molecular entities.

The differential diagnosis of DLB is further complicated by overlap with Alzheimer’s disease (AD), which is primarily characterized by amyloid-β and tau pathology [3]. Mixed AD and Lewy body pathologies are common, and clinical phenotypes may converge across disease stages. These overlaps complicate disease-specific molecular classification and motivate direct disease–disease comparisons.

Genetic studies further support a heterogeneous LBD architecture involving shared and disease-associated loci and pathways related to synuclein, lysosomal, lipid, immune, and broader neurodegenerative biology [4,5].

Recent integrative studies have expanded this view. Reviews summarize recurrent involvement of *SNCA*, *GBA1*, and *APOE* and pathways related to protein degradation, neuroinflammation, mitochondrial dysfunction, and cellular stress [6]. Zhu et al. integrated a large LBD GWAS meta-analysis with functional genomic datasets and prioritized 85 candidate LBD-associated genes [7]. These data provide an external framework for prioritizing transcriptomic findings.

Transcriptomic analysis can reveal disease-associated molecular changes in affected tissue, but bulk brain profiles may reflect regulation within surviving cells, neuronal loss, gliosis, shifts in cell-type composition, postmortem factors, and disease stage. Consequently, direct comparisons among DLB, PDD, and AD require stringent multiple-testing control and cautious biological interpretation.

GSE150696 is informative because it includes postmortem prefrontal cortex samples from controls and individuals with AD, DLB, and PDD within a single dataset. Xu et al. previously analyzed GSE150696 using differential-expression, pathway, protein–protein interaction, and co-expression network approaches [8]. However, the previous and present studies use materially different analytical pipelines; their candidate lists should therefore be interpreted as complementary rather than as direct replication or contradiction.

Single-nucleus transcriptomics provides complementary cell-type context for bulk-tissue findings. Feleke et al. analyzed paired bulk and single-nucleus RNA sequencing in Lewy body diseases and demonstrated both shared and cell-type-specific transcriptional abnormalities across neuronal and glial populations [9].

Accordingly, the present study reanalyzed GSE150696 using gene-symbol-level summarization, age- and sex-adjusted empirical-Bayes models, transcriptome-wide BH-FDR correction, and prespecified sensitivity analyses. Post hoc cross-disease candidates were kept separate from FDR-supported findings, compared with LBD genes prioritized by Zhu et al. [7], and interpreted using cell-type-resolved evidence from GSE178146 [9]. Unlike previous analyses of GSE150696, this framework explicitly combines rigorous multiplicity control, model-robustness assessment, prospective power and sample-size evaluation, external genetic prioritization, and cell-type context while maintaining a hierarchy between confirmatory and exploratory evidence.

## 2. Results

### 2.1. Analytical Workflow and Discovery Cohort

**Figure 1:**
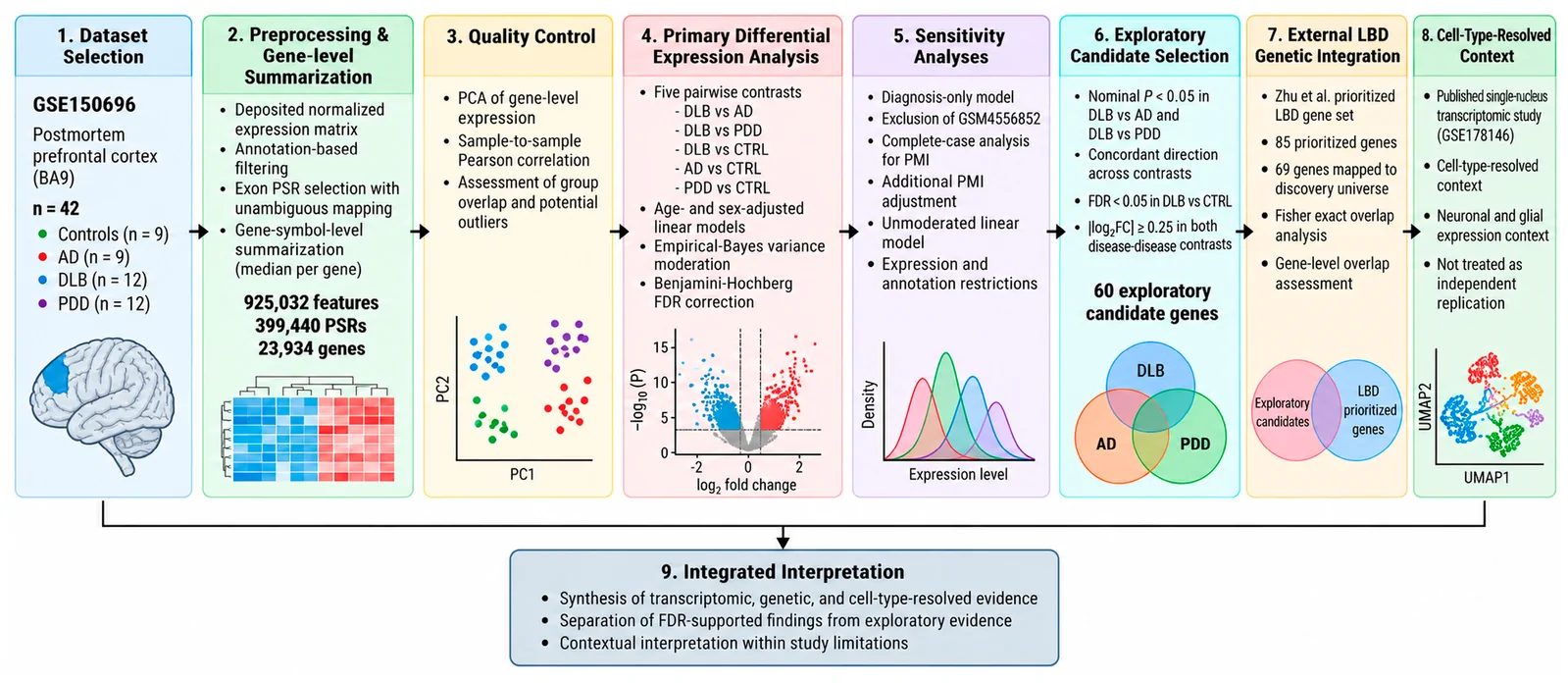
Analytical workflow of the study. The workflow proceeded sequentially from dataset selection and gene-level preprocessing to quality-control assessment, primary age- and sex-adjusted differential-expression analysis, sensitivity analyses, post hoc exploratory candidate selection, external LBD genetic/multi-omics overlap analysis, cell-type-resolved contextualization using GSE178146, and integrated interpretation.Black arrows indicate the sequential progression of the analytical workflow and its convergence into the integrated interpretation step. The pastel panel background colors are used only to visually distinguish the analytical stages and do not represent statistical significance or effect magnitude. Where diagnostic groups are color-coded, green, red, blue, and purple denote CTRL, AD, DLB, and PDD, respectively. Abbreviations: AD, Alzheimer’s disease; CTRL, neurologically unaffected control; DLB, dementia with Lewy bodies; PDD, Parkinson’s disease dementia; LBD, Lewy body dementia; QC, quality control; PCA, principal component analysis; PSR, probe selection region; PMI, postmortem interval; FDR, false discovery rate; UMAP, uniform manifold approximation and projection. Figure 1 was prepared by the authors using BioRender (BioRender.com) and is available at the following project link: https://app.biorender.com/illustrations/6a4be0d5ccb2b68499c5be32.

The overall analytical strategy is summarized in Figure 1. The primary discovery analysis was based on the GSE150696 prefrontal cortex dataset [10], comprising 42 postmortem samples: nine neurologically unaffected controls, nine individuals with Alzheimer’s disease (AD), 12 with dementia with Lewy bodies (DLB), and 12 with Parkinson’s disease dementia (PDD). Mean ages were 84.67 ± 6.24 years in controls, 85.67 ± 6.75 years in AD, 80.17 ± 7.37 years in DLB, and 78.08 ± 6.26 years in PDD. The female-to-male distribution was 5:4 in both the control and AD groups and 6:6 in both the DLB and PDD groups.

The deposited expression matrix contained 925,032 transcriptomic features. After annotation-based filtering, 399,440 exon probe-selection-region features with unambiguous gene and transcript-cluster assignments were retained. Median summarization at the gene-symbol level generated expression profiles for 23,934 unique genes, which constituted the discovery expression universe used for subsequent differential-expression and integrative analyses.

The analytical framework consisted of gene-level expression preprocessing and quality assessment, differential-expression analysis across five diagnostic contrasts, sensitivity analyses of the principal findings, post hoc definition of an exploratory cross-disease candidate set, integration with published LBD genetic evidence [7], and interpretation in the context of published cell-type-resolved transcriptomic findings from GSE178146 [9] (Figure 1).

The workflow comprised preprocessing and gene-level summarization of GSE150696, quality-control assessment, differential-expression analysis across DLB, AD, PDD, and neurologically unaffected controls, sensitivity analyses, post hoc definition of an exploratory cross-disease candidate set, integration with published LBD genetic and multi-omics evidence, and interpretation in the context of published cell-type-resolved transcriptomic findings from GSE178146.

### 2.2. Global Expression Structure and Differential Expression Across Diagnostic Groups

**Figure 2:**
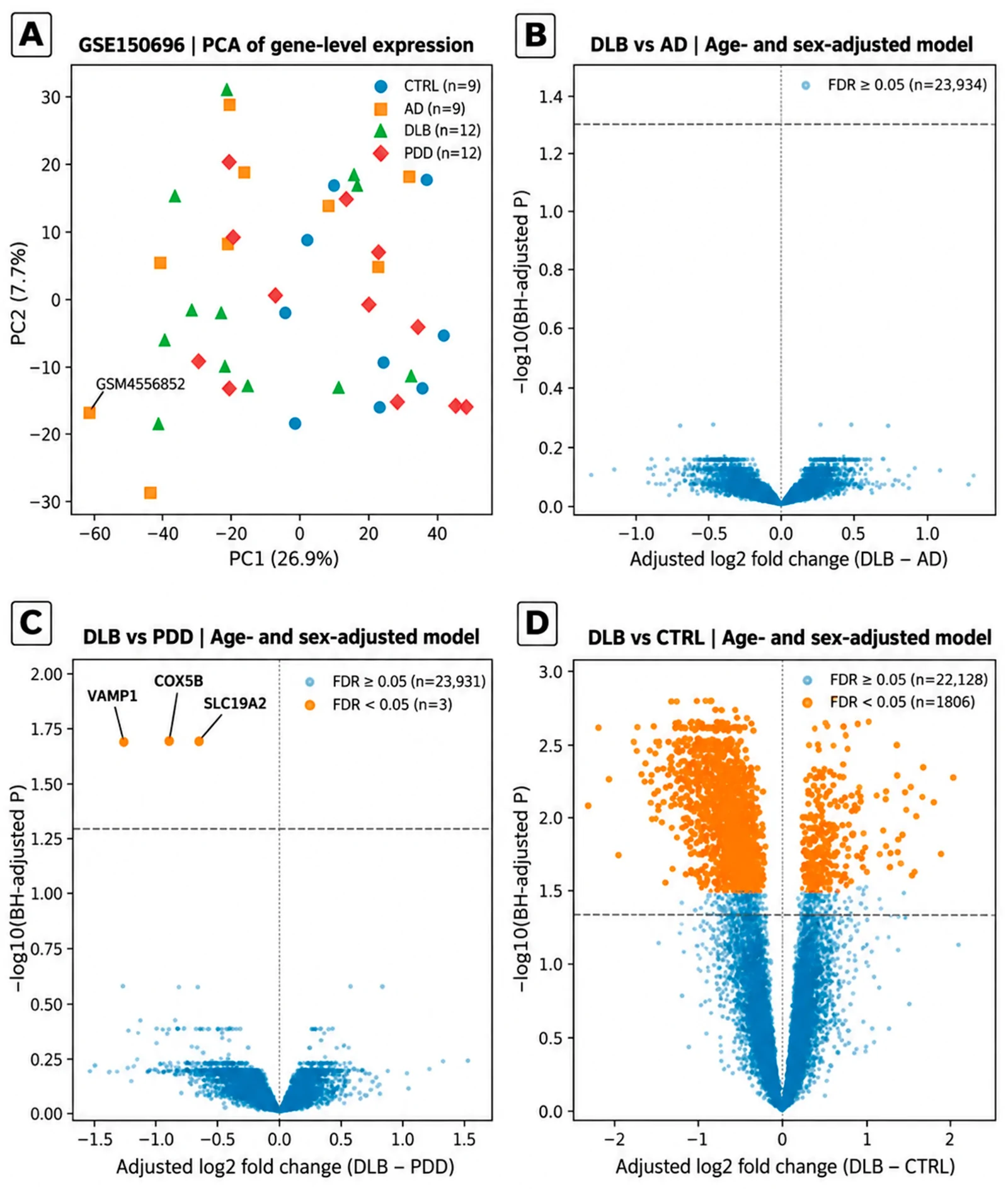
Gene-level transcriptomic findings in GSE150696. (**A**) Principal component analysis of the 23,934-gene expression matrix across control, AD, DLB, and PDD samples. (**B**) Volcano plot of the DLB-versus-AD comparison; no gene reached BH-FDR < 0.05. (**C**) Volcano plot of the DLB-versus-PDD comparison highlighting the three genes FDR-supported in the primary age- and sex-adjusted model:*SLC19A2*,*VAMP1*, and*COX5B*. (**D**) Volcano plot of the DLB-versus-control comparison, which demonstrated substantially broader transcriptomic alterations. In panels (**B**–**D**), the horizontal dashed line indicates the BH-FDR significance threshold of 0.05, and the vertical dotted line indicates log2FC = 0. Positive DLB-centered log2 fold changes indicate higher expression in DLB, whereas negative values indicate lower expression in DLB. Abbreviations: AD, Alzheimer’s disease; CTRL, neurologically unaffected control; DLB, dementia with Lewy bodies; PDD, Parkinson’s disease dementia; PCA, principal component analysis; BH-FDR, Benjamini–Hochberg false discovery rate; log2FC, log2 fold change.

Principal component analysis of the gene-level expression matrix showed substantial overlap among the four diagnostic groups (Figure 2A). The first principal component accounted for 26.86% of the total expression variance, whereas the second principal component accounted for 7.71%. No clearly separated disease-specific clusters were evident, indicating considerable global transcriptomic overlap among the diagnostic groups. Median Pearson correlations between each sample and the remaining samples ranged from 0.9533 to 0.9757, indicating generally high sample-to-sample similarity. GSM4556852, an AD sample, showed the lowest median correlation (0.9533); this sample was retained in the primary analysis and evaluated separately in an exclusion-based sensitivity analysis. In the primary age- and sex-adjusted analysis, no gene reached FDR < 0.05 in the DLB-versus-AD comparison (Figure 2B). The minimum adjusted *p*-value was 0.5549, indicating an absence of transcriptome-wide significant single-gene differences between DLB and AD under the primary model.

In the primary age- and sex-adjusted analysis, three genes—*SLC19A2*, *VAMP1*, and *COX5B*—were identified as significantly differentially expressed between DLB and PDD after transcriptome-wide FDR correction (Figure 2C). All three showed lower expression in DLB relative to PDD. *SLC19A2* exhibited a log2 fold change of −0.670 (95% CI, −0.912 to −0.427; *p* = 1.76 × 10^−6^; FDR = 0.02064), *VAMP1* a log2 fold change of −1.299 (95% CI, −1.771 to −0.828; *p* = 1.83 × 10^−6^; FDR = 0.02064), and *COX5B* a log2 fold change of −0.869 (95% CI, −1.191 to −0.547; *p* = 2.59 × 10^−6^; FDR = 0.02064). The most extensive differential-expression pattern was observed in the DLB-versus-control comparison (Figure 2D).

A total of 1806 genes reached FDR < 0.05, including 307 with higher and 1499 with lower expression in DLB. Among these, 1304 additionally exhibited |log2FC| ≥ 0.5. The AD-versus-control comparison identified 612 FDR-supported genes, comprising 105 genes with higher and 507 with lower expression in AD, whereas no gene reached FDR < 0.05 in the PDD-versus-control comparison. The overall differential-expression findings are summarized in Table 1.

Overall, the transcriptomic difference between DLB and neurologically unaffected controls was considerably broader than the direct disease–disease differences. By comparison, direct differentiation of DLB from PDD was restricted to three genes that were FDR-supported in the primary age- and sex-adjusted model, whereas no FDR-supported gene was detected between DLB and AD.

### 2.3. Cross-Disease Evaluation of the DLB-Versus-PDD Candidate Genes

The three genes FDR-supported in the primary age- and sex-adjusted DLB-versus-PDD model were further evaluated across the remaining diagnostic contrasts. All three also showed lower expression in DLB relative to controls. The corresponding adjusted *p*-values were 0.01193 for SLC19A2, 0.04159 for VAMP1, and 0.03871 for COX5B.

Among these genes, *SLC19A2* additionally showed nominal evidence of lower expression in DLB than in AD (log2FC = −0.341, *p* = 0.01506), although the association did not remain significant after transcriptome-wide multiple-testing correction (FDR = 0.7674). *VAMP1* showed a DLB-versus-AD log2FC of −0.218 (*p* = 0.4099, FDR = 0.9296), whereas *COX5B* showed a log2FC of −0.296 (*p* = 0.1047, FDR = 0.8628).

The cross-comparison results for these three genes are summarized in Table 2.

### 2.4. Robustness of the DLB-Versus-PDD Associations

The stability of the three principal DLB-versus-PDD candidates was examined across alternative model specifications and sample subsets (Figure S1). *SLC19A2*, *VAMP1*, and *COX5B* remained FDR-supported in the diagnosis-only model and after exclusion of GSM4556852, the sample exhibiting the lowest median correlation in the quality-control analysis. When the analysis was restricted to the 41 individuals with complete postmortem interval information, all three genes also remained significant in the age- and sex-adjusted model, with FDR = 0.04356 for each candidate.

However, additional adjustment for postmortem interval within the same 41-sample subset attenuated the statistical evidence. Under this model, the adjusted *p*-values increased to 0.12095 for *SLC19A2* and *VAMP1* and to 0.18140 for *COX5B*. Importantly, the direction of effect remained unchanged for all three genes. The findings were also sensitive to the variance-model specification. In an unmoderated ordinary linear-model analysis, none of the three genes remained below the FDR threshold of 0.05, although they remained among the strongest DLB-versus-PDD signals, with adjusted *p*-values of approximately 0.052.

Additional expression- and annotation-based sensitivity analyses were performed. Restriction to genes with mean log2 expression >5 retained all three candidates, as did restriction to genes whose contributing probe sets mapped to a single transcript cluster (Figure S1). In the DLB-versus-control contrast, no gene reached FDR < 0.05 in the diagnosis-only model, compared with 1806 genes in the primary age- and sex-adjusted model and 1705 genes in the complete-case model additionally adjusted for PMI.

### 2.5. Exploratory Cross-Disease Candidate Set

No individual gene reached FDR < 0.05 in both the DLB-versus-AD and DLB-versus-PDD comparisons while maintaining a concordant direction of effect. Accordingly, the discovery analysis did not identify any gene with transcriptome-wide FDR-supported evidence for differentiating DLB from both AD and PDD. Given the absence of a gene satisfying these stringent criteria, a separately defined post hoc exploratory analysis was performed to identify genes showing consistent, although weaker, evidence of DLB-associated expression differences across disease comparisons.

Genes were required to show nominal *p* < 0.05 in both the DLB-versus-AD and DLB-versus-PDD comparisons, concordant directions of effect across these two disease–disease contrasts, and FDR < 0.05 in the DLB-versus-control comparison. Application of these criteria identified 79 genes. An additional requirement of |log2FC| ≥ 0.25 in both disease–disease comparisons reduced the final exploratory candidate set to 60 genes. Seven of these genes showed higher expression in DLB, whereas 53 showed lower expression. All 60 candidates exhibited expression changes in the same direction in DLB relative to controls. Their cross-comparison expression patterns are presented in Figure S2.

Among the exploratory candidates were *SLC29A4*, *PTS*, *THOC7*, *ADSS*, *TUBB4B*, *KCTD8*, *NXPE2*, and *SLC19A2*. Notably, *SLC19A2* was also one of the three genes FDR-supported in the primary age- and sex-adjusted DLB-versus-PDD comparison. However, most disease–disease associations within the 60-gene set did not remain statistically significant after transcriptome-wide multiple-testing correction. Accordingly, the 60-gene set was interpreted as a hypothesis-generating group of cross-disease candidates rather than as a validated DLB-discriminatory molecular signature.

### 2.6. Integration with Published LBD Genetic Evidence

The exploratory transcriptional candidates were subsequently evaluated for overlap with published LBD genetic and multi-omics prioritization evidence. The 60-gene exploratory set and the three candidates FDR-supported in the primary age- and sex-adjusted DLB-versus-PDD model were compared with the 85 genes prioritized in the GWAS and multi-omics study by Zhu et al. [7]. Of the 85 reference genes, 69 could be mapped to the 23,934-gene GSE150696 discovery universe, including 62 exact gene-symbol matches and seven curated alias mappings. Sixteen genes remained unresolved.

None of the three candidates FDR-supported in the primary age- and sex-adjusted DLB-versus-PDD model—*SLC19A2*, *VAMP1*, and *COX5B*—overlapped with the external 85-gene LBD-prioritized set. In contrast, *SYT11* was the only gene shared between the 60-gene exploratory transcriptional set and the LBD genetic reference set. The overlap between the 60-gene exploratory set and the mapped LBD-prioritized genes was not statistically significant in the descriptive two-sided Fisher exact analysis (*p* = 0.1592). Following correction across the five evaluated overlap tests, the BH-adjusted *p*-value was 0.3404. Thus, the exploratory transcriptional candidate set did not show statistically significant evidence of overall enrichment for the published LBD-prioritized genes.

*SYT11* was the sole exploratory overlap between the 60-gene transcriptomic candidate set and the mapped LBD-prioritized gene set. This overlap was hypothesis-generating and was not supported by significant enrichment of the broader candidate set. Within the external prioritization framework [7], *SYT11* was supported by GWAS significance and gene-mapping evidence and also received support from gene-set enrichment, drug–gene interaction, and gene-expression components. However, positive evidence was not reported for the gene-based, TWAS, colocalization, SMR, or PWAS categories. Within GSE150696, *SYT11* exhibited consistently lower expression in DLB across the relevant comparisons. The DLB-versus-AD contrast showed log2FC = −0.506 (*p* = 0.04035, FDR = 0.8332), whereas DLB versus PDD showed log2FC = −0.559 (*p* = 0.01229, FDR = 0.5915). In contrast, the DLB-versus-control association was transcriptome-wide significant (log2FC = −1.371, FDR = 0.00301).

The integrated evidence for *SLC19A2*, *VAMP1*, *COX5B*, and *SYT11* is summarized in Table 3. The first three genes were principally supported by direct DLB-versus-PDD transcriptomic evidence, whereas *SYT11* was the sole exploratory overlap between the transcriptomic candidate list and the published LBD-prioritized gene set; this overlap was not supported by significant enrichment and requires independent validation.

### 2.7. Cell-Type-Resolved Transcriptomic Evidence from GSE178146 

The bulk-tissue results were further interpreted in relation to the published single-nucleus transcriptomic study corresponding to GSE178146 by Feleke et al. [9]. The study analyzed anterior cingulate cortex tissue from 28 individuals, including seven neurologically unaffected controls and seven individuals each with Parkinson’s disease, PDD, and DLB. The published single-nucleus analysis demonstrated extensive transcriptional alterations across neuronal and non-neuronal cell populations. DLB and PDD showed substantial overall molecular similarity; nevertheless, direct DLB-versus-PDD comparisons revealed cell-type-resolved differences. Across the reported cell-type-specific comparisons, 303 genes were downregulated and 87 were upregulated in DLB relative to PDD [9].

The published findings further showed that transcriptional alterations were not uniformly distributed across cell types, with prominent changes particularly evident in excitatory neuronal populations and oligodendrocytes. These observations provide a cellular context for the comparatively restricted number of robust bulk-tissue differences detected between DLB and PDD in the present analysis. GSE178146 was not treated as a formal candidate-specific replication dataset because donor-level single-nucleus data were not independently reanalyzed in the present study. Furthermore, expression information derived from GSE178146 contributed to the multi-omics framework of Zhu et al. [7]. Consequently, the two evidence sources were not considered statistically independent validation layers. This distinction is reflected in the evidence classification summarized in Table 3.

### 2.8. Integrated Summary of Transcriptomic and Genetic Evidence

The transcriptomic, sensitivity, genetic, and cell-type-resolved analyses identified two complementary evidence categories (Table 3). *SLC19A2*, *VAMP1*, and *COX5B* were the three genes FDR-supported in the primary age- and sex-adjusted DLB-versus-PDD model and showed lower expression in DLB relative to controls (Table 2). Their effect directions were preserved across the principal sensitivity analyses, while adjusted significance was attenuated after PMI adjustment and in the unmoderated linear-model analysis (Figure S1).

*SYT11* was the sole gene shared between the 60-gene exploratory transcriptomic set and the published LBD-prioritized gene set. *SYT11* showed lower expression across the relevant DLB-centered contrasts, while the broader 60-gene set did not show statistically significant enrichment for the external LBD genetic set.

The 60-gene exploratory set showed concordant cross-disease expression patterns (Figure S2), whereas the external overlap analysis identified only the single *SYT11* overlap described above.

### 2.9. Statistical Power and Prospective Replication Sample-Size Estimates

At a conventional two-sided α = 0.05, the current DLB-versus-PDD comparison (12 versus 12 samples) provides 80% power only for standardized effects of approximately Cohen’s d ≥ 1.20 and 90% power for d ≥ 1.39. For DLB versus AD (12 versus nine samples), the corresponding detectable effects are approximately d ≥ 1.30 and d ≥ 1.51, respectively. Thus, the present design is mainly sensitive to large disease–disease effects and has limited sensitivity for moderate effects; transcriptome-wide FDR control would impose an additional multiplicity burden beyond these conventional calculations.

Using the primary-model effect estimates and nominal moderated confidence intervals, the approximate observed standardized effects for SLC19A2, VAMP1, and COX5B were d = 2.21, 2.20, and 2.16, respectively. With α = 0.0167, use of the unshrunk discovery effects yielded estimated requirements of six, six, and seven samples per group for 80% power and eight samples per group for 90% power. Because effect sizes estimated in a small discovery cohort may be upwardly biased, these values were considered optimistic. After applying a 50% shrinkage for conservative replication planning, the anticipated effects were d = 1.11, 1.10, and 1.08. Under the same α = 0.0167, the estimated sample sizes required per group for 80% power were 19, 19, and 20, respectively; for 90% power, the corresponding estimates were 24, 24, and 25. Accordingly, a targeted independent replication study would require approximately 20 DLB and 20 PDD samples for 80% power, or approximately 25 samples per group for 90% power, under these conservative planning assumptions.

## 3. Discussion

In the present study, we used an integrative genetic and transcriptomic framework to investigate molecular differences among DLB, AD, and PDD. Three principal observations emerged. First, direct disease–disease transcriptomic separation was limited relative to disease–control differences, and no individual gene reached transcriptome-wide FDR significance in both the DLB-versus-AD and DLB-versus-PDD comparisons. Second, *SLC19A2*, *VAMP1*, and *COX5B* represented the strongest direct DLB-versus-PDD differences; however, none simultaneously reached transcriptome-wide FDR significance in both disease–disease contrasts. Their effect directions and magnitudes were relatively stable across several sensitivity analyses, whereas formal statistical significance was partly dependent on covariate and variance-model specification. Third, *SYT11* was the sole exploratory overlap between the post hoc transcriptomic set and the LBD-prioritized genes reported by Zhu et al. [7], without significant enrichment of the broader candidate set. Overall, the global expression structure and limited robust single-gene separation are compatible with molecular overlap, but the small cohort and model-dependent significance preclude interpretation as biological equivalence.

The limited transcriptomic separation between DLB and PDD is consistent with their close clinical, pathological, and genetic relationships. Although the distinction between DLB and PDD remains clinically important, it is primarily defined by the temporal relationship between dementia and parkinsonism rather than by sharply divergent neuropathological criteria [1,2]. Genomic studies have similarly demonstrated overlapping susceptibility with Parkinson’s disease and AD [4,5,7]. In particular, the recent LBD GWAS meta-analysis by Zhu et al. reported positive genetic correlations of LBD with both AD and Parkinson’s disease, further supporting a partially shared etiological architecture [7]. The substantial overlap observed in our PCA and the absence of any gene satisfying FDR < 0.05 simultaneously in both DLB-versus-AD and DLB-versus-PDD comparisons are therefore compatible with the broader concept of molecular continuity across neurodegenerative dementias rather than strict molecular separation.

Comparison with the previous analysis of GSE150696 by Xu et al. [8] requires attention to methodological differences. Xu et al. used GEO2R with nominal significance and effect-size filtering followed by network analyses, whereas the present study used gene-symbol-level summarization and transcriptome-wide BH correction, with effect-size thresholds restricted to predefined descriptive or exploratory analyses. These differences are expected to generate different candidate lists; accordingly, the two studies should be viewed as complementary analyses of the same source dataset rather than as direct replication or contradiction.

Among the three genes FDR-supported in the primary age- and sex-adjusted DLB-versus-PDD model, *SLC19A2* is biologically notable because it encodes thiamine transporter 1. Recent structural and functional work confirms that SLC19A2, together with SLC19A3, mediates cellular thiamine uptake and links thiamine transport to cellular energy metabolism and neurometabolic disease [11]. In the present dataset, *SLC19A2* was lower in DLB than in PDD and controls and also showed nominal evidence of lower expression relative to AD. These findings may be compatible with altered thiamine-related cortical metabolic processes in DLB; however, bulk BA9 data cannot determine whether the observed difference reflects cell-intrinsic transcriptional regulation, neuronal loss, altered cellular composition, or disease-stage effects. Cell-type-resolved and functional studies will therefore be required, and the model sensitivity of the association argues against interpreting *SLC19A2* as a disease-specific biomarker at this stage.

*VAMP1* provides a second biologically plausible candidate. VAMP1 is a vesicle-associated SNARE protein that contributes to synaptic vesicle fusion and neurotransmitter release [12]. Altered synaptic vesicle cycling and membrane trafficking are increasingly recognized as features of synucleinopathies, and multiple Parkinson’s disease-associated proteins—including α-synuclein and proteins involved in autophagy and vesicle recycling—intersect with these pathways [13]. The observed *VAMP1* expression pattern may therefore be compatible with altered synaptic or vesicular processes in DLB, although bulk-tissue data cannot establish whether this reflects regulation within surviving neurons or differences in cellular composition. Xu et al. also identified vesicle localization and synaptic-related processes in the same GSE150696 cohort [8], supporting pathway-level follow-up despite differences in candidate-gene selection.

The third gene FDR-supported in the primary age- and sex-adjusted model, *COX5B*, encodes the nuclear-encoded Vb subunit of cytochrome c oxidase (complex IV), the terminal enzyme complex of the mitochondrial respiratory chain. Contemporary reviews of cytochrome c oxidase describe subunit Vb as a nuclear-encoded component of complex IV and emphasize the central role of complex IV regulation in oxidative phosphorylation, ATP production, and redox homeostasis [14]. Mitochondrial dysfunction and impaired oxidative phosphorylation are also well established across neurodegenerative disorders [15]. The lower *COX5B* expression may therefore be compatible with altered mitochondrial processes in DLB, but bulk postmortem tissue cannot distinguish intrinsic regulation from neuronal loss, cellular composition, or disease-stage effects.

The genetic–transcriptomic integration provided a different level of evidence. None of the three primary DLB-versus-PDD candidates overlapped with the 85 LBD-associated genes prioritized by Zhu et al. [7], and the overall overlap between the 60-gene exploratory set and the external *LBD* gene set was not statistically significant. This absence of broad enrichment is informative. Germline genetic association and postmortem gene expression measure fundamentally different aspects of disease biology: inherited susceptibility may act upstream of numerous downstream molecular events, whereas brain transcriptomic profiles reflect a combination of disease progression, cellular responses, neuronal loss, inflammation, treatment exposure, and tissue composition. Consequently, strong genome-wide overlap between genetically prioritized genes and disease-state differentially expressed genes should not necessarily be expected. The lack of significant enrichment therefore argues against claiming general genetic validation of the 60-gene set and supports our decision to retain it as hypothesis-generating.

Within this context, *SYT11* was the sole exploratory overlap between the 60-gene transcriptomic candidate set and the LBD-prioritized gene set of Zhu et al. [7]. Because the broader candidate set was not significantly enriched for LBD-prioritized genes, this individual overlap should be regarded as hypothesis-generating rather than as genetic validation. In the present dataset, *SYT11* showed consistently lower expression in DLB relative to AD, PDD, and controls, although only the DLB-versus-control association survived transcriptome-wide correction. Experimental evidence provides biological plausibility for involvement of *SYT11* in synucleinopathy-related mechanisms. Bento et al. demonstrated that *SYT11* functions within an *ATP13A2*-related pathway regulating lysosomal function and autophagosome degradation, with reduced *SYT11* contributing to lysosomal dysfunction [16]. Conversely, Wang et al. showed that excessive synaptotagmin-11 can impair vesicle replenishment and dopamine release and mediate parkin-associated neurotoxicity in experimental models [17]. Together, these observations suggest that the relationship between *SYT11* abundance and neuronal function may be context-dependent rather than simply directional. Our finding of reduced cortical *SYT11* expression should therefore not be interpreted mechanistically as equivalent to experimental overexpression or depletion models. Instead, the present observation identifies *SYT11* as a candidate for independent follow-up because it combines an exploratory expression pattern with membership in an external LBD-prioritized list.

The cell-type-resolved study of Feleke et al. provides an additional framework for interpreting the relatively modest bulk-tissue DLB–PDD differences [9]. Their paired bulk and single-nucleus analyses showed substantial transcriptional similarity between DLB and PDD while also identifying cell-type-specific abnormalities, including changes in RNA processing and splicing. Such signals can be attenuated when expression is averaged across heterogeneous cortical tissue. Moreover, the gene-level summarization used here was designed to evaluate overall gene expression and did not retain transcript- or isoform-level information, so biologically relevant splicing differences may remain undetected.

The absence of FDR-supported genes in the DLB-versus-AD comparison should likewise not be interpreted as evidence that the two disorders are transcriptionally indistinguishable. A substantially larger neuropathologically defined study by Olney et al. identified both shared and distinct gene-expression abnormalities in LBD and AD [18]. Differences in cohort size, brain region, pathological classification, platform, and statistical power are likely to contribute to the differing results. The design-sensitivity analysis confirmed that the current DLB-versus-AD comparison is mainly powered to detect large effects, so moderate differences may remain undetected, particularly after transcriptome-wide multiplicity control. The negative FDR result is therefore best interpreted as an absence of robust single-gene evidence within this dataset rather than as evidence of biological equivalence.

Recent omics studies further emphasize that Lewy body dementia has a heterogeneous molecular architecture across data modalities. Reviews integrating genomic, epigenomic, transcriptomic, and proteomic evidence have highlighted extensive shared biology between DLB and PDD while also identifying potentially disease- and stage-associated molecular signatures [19,20]. Network proteomic analysis of LBD cortex has identified presynaptic, metabolic, inflammatory, and extracellular-matrix modules that differ from AD and vary with concomitant amyloid burden, while comparative proteomics across AD, DLB, and PDD has identified both shared modules and disorder-associated proteins [21,22]. Genetic studies have likewise demonstrated heterogeneity within Lewy body disease, including genetic effects associated with dementia status, sex-specific X-chromosomal signals, and population-specific risk loci [23,24,25]. Polygenic-risk analyses further suggest that aggregate genetic information may contribute to discrimination of LBD from AD [26], and recent plasma proteomic studies have identified both shared and differential biomarker patterns across dementia syndromes [27]. Broader GWAS syntheses also emphasize common pathways across dementia subtypes, including lipid metabolism, immune responses, lysosomal mechanisms, and cellular homeostasis [28]. These findings reinforce the need to interpret bulk-tissue gene-expression differences as one layer within a broader multi-omics and clinically heterogeneous disease framework.

Control comparisons also require cautious interpretation because the number of FDR-supported discoveries changed substantially across model specifications. The broad DLB-versus-control signal in the age- and sex-adjusted model was not reproduced in the diagnosis-only analysis, indicating sensitivity to covariate structure. Likewise, the absence of FDR-supported PDD-versus-control genes contrasts with published cell-type-resolved evidence of PDD-associated transcriptional abnormalities [9]. These patterns reinforce the importance of effect direction, model stability, and cross-dataset biological context rather than reliance on discovery counts alone.

The sensitivity analyses are therefore central to interpretation of the candidate genes. *SLC19A2*, *VAMP1*, and *COX5B* retained their effect directions across alternative specifications and remained significant in several sensitivity models, but their FDR significance was lost after additional PMI adjustment and in the unmoderated ordinary linear model. Collectively, the direction and magnitude of these expression differences were relatively stable, whereas formal statistical significance was partly dependent on covariate and variance-model specification. Replication in larger neuropathologically characterized cohorts is necessary before these genes can be regarded as robust DLB–PDD discriminators.

The exploratory 60-gene set should be interpreted within the same framework. This set was intentionally derived after the primary FDR-controlled analysis failed to identify a gene significantly distinguishing DLB from both AD and PDD. Its purpose was therefore candidate prioritization rather than statistical discovery. The requirement for concordant direction across both disease–disease contrasts, nominal evidence in each comparison, significant DLB-versus-control association, and an additional effect-size threshold reduced the candidate space and identified genes showing internally coherent patterns. Nevertheless, because these criteria were selected post hoc, they do not correct for the selection process itself and cannot confer confirmatory significance. The absence of significant enrichment against the external 85-gene LBD reference set further supports retaining the 60-gene panel as hypothesis-generating.

This study has several strengths. The analysis incorporated all four diagnostic groups from a single cortical dataset, enabling direct comparison of DLB with both AD and PDD under a common preprocessing and statistical framework. Transcriptome-wide multiple-testing correction was applied consistently, and primary findings were subjected to multiple sensitivity analyses rather than being interpreted from a single model. In addition, genetic integration was used to distinguish transcriptomic association from exploratory overlap with external genetic prioritization, while GSE178146 was deliberately treated as cell-type-resolved context rather than counted as an independent replication dataset because it also contributed to the expression evidence incorporated into the Zhu et al. framework [7,9]. This conservative evidence hierarchy reduces the risk of double-counting related data sources.

Several limitations should also be acknowledged. First, the discovery cohort was modest, particularly for AD and control groups, limiting statistical power for direct disease–disease comparisons. The design-sensitivity analysis confirmed that the present contrasts are primarily powered to detect large standardized effects, whereas moderate differences may remain undetected; the prospective replication estimates should therefore be viewed as planning targets derived from discovery-cohort effects rather than guarantees. Second, expression was derived from postmortem bulk prefrontal cortex, and cellular composition could not be directly modeled. Third, PMI was missing for one participant, and additional PMI adjustment attenuated the principal DLB-versus-PDD associations. Fourth, the analysis summarized exon-level measurements to gene-level medians and consequently did not evaluate alternative transcript usage or splicing, despite evidence that RNA-splicing dysregulation is prominent in Lewy body dementias [9]. Fifth, the 60-gene cross-disease set was derived post hoc and should not be considered a validated molecular signature. Sixth, the genetic overlap analyses were descriptive and did not account for all factors that can influence gene prioritization, including locus structure, correlated genes, or differential probability of gene representation across platforms. Finally, no fully independent cohort with equivalent DLB, PDD, AD, and control groups was available for direct replication of the candidate genes.

Future studies should determine whether the observed candidate-level differences reflect reproducible disease-related molecular alterations, cell-type-specific effects, or differences in tissue composition and disease stage. Larger, neuropathologically characterized cohorts integrating single-cell or single-nucleus multi-omics, transcript- and isoform-level profiling, proteogenomics, and functional validation will be needed to clarify the biological relevance of these findings and to support robust integrative modeling.

## 4. Materials and Methods

### 4.1. Study Design and Analytical Framework

This study was designed as an integrative secondary analysis of publicly available transcriptomic and genetic data to investigate molecular differences among DLB, AD, and PDD. The analytical workflow comprised three complementary components. First, bulk brain transcriptomic data from GSE150696 were reanalyzed at the gene level to characterize differential expression across DLB, AD, PDD, and neurologically unaffected control groups. Second, transcriptomic findings were integrated with genes prioritized in a recent large-scale LBD genome-wide association and multi-omics study [7]. Third, the results were interpreted in the context of published cell-type-resolved transcriptomic findings from GSE178146 [9].

The primary disease–disease comparisons were DLB versus AD and DLB versus PDD. DLB-versus-control, AD-versus-control, and PDD-versus-control contrasts were additionally examined to distinguish disease-discriminatory signals from broader disease-associated expression changes. Primary transcriptome-wide findings, sensitivity analyses, and post hoc exploratory candidate analyses were treated as separate levels of evidence throughout the study.

### 4.2. Discovery Transcriptomic Dataset and Clinical Metadata

The primary transcriptomic dataset was obtained from the Gene Expression Omnibus (GEO) accession GSE150696 [10]. The dataset contains postmortem prefrontal cortex tissue from Brodmann area 9 profiled using the GeneChip® Human Transcriptome Array 2.0 (Affymetrix, Inc., Santa Clara, CA, USA). A total of 42 samples were included: 9 neurologically unaffected controls, 9 individuals with AD, 12 with DLB, and 12 with PDD.

Age and sex information was available for all 42 individuals. Postmortem interval (PMI) information was available for 41 samples and was missing for one PDD sample (GSM4556883). Diagnostic group, age, sex, and PMI information were linked to expression measurements using the corresponding GEO sample identifiers. The deposited expression matrix and associated annotation information were used for the present secondary analysis. No new biological samples were generated or experimentally processed, and no chemicals, reagents, laboratory instruments, commercial cell lines, or newly acquired biological materials were directly used in the present secondary analysi.

### 4.3. Expression Data Preprocessing and Gene-Level Summarization

The deposited GSE150696 expression data had been preprocessed by the original investigators using Partek Genomics Suite® version 7.0 (Partek Inc., St. Louis, MO, USA), as reported in the GEO record [10]. Source-level preprocessing included robust multi-array average (RMA) background correction, quantile normalization, log2 transformation, and median-polish summarization. In the present secondary analysis, raw CEL files were not reprocessed. Instead, the normalized expression matrix deposited in GEO was used as the starting point for subsequent annotation filtering and gene-level summarization.

The deposited GSE150696 matrix contained 925,032 transcriptomic features across the 42 samples. The present analysis focused on exon probe-selection-region (PSR) features with unambiguous assignments to an individual gene symbol and transcript cluster. Features representing junctions, control probes, unannotated features, or ambiguous gene assignments were excluded. Following annotation filtering, 399,440 exon PSRs were retained. For each sample, the normalized log2 expression values of retained exon PSRs assigned to the same gene symbol were summarized using the median. This additional gene-level summarization generated expression profiles for 23,934 unique gene symbols, with no missing expression values in the resulting matrix. Importantly, this procedure represented an additional gene-symbol-level summarization of the already normalized deposited measurements and should not be interpreted as a new RMA preprocessing or transcript-cluster-level normalization from raw CEL files.

A total of 455 gene symbols had contributing PSRs assigned to more than one transcript cluster. These genes were retained in the primary analysis to avoid removing otherwise informative expression profiles, and the robustness of the main findings was subsequently examined after restricting the dataset to gene symbols associated with a single transcript cluster.

### 4.4. Gene-Level Quality-Control Assessment

Quality-control assessment was performed on the summarized 23,934-gene expression matrix before differential-expression testing. Global expression variation was examined using principal component analysis (PCA). Before PCA, the expression profile of each gene was mean-centered across samples; no unit-variance scaling or covariate residualization was applied. PCA was performed on the resulting centered sample-by-gene expression matrix, and the first two principal components were examined to assess global sample structure, diagnostic-group overlap, and potential outlying samples.

Sample-to-sample similarity was additionally assessed using pairwise Pearson correlation coefficients calculated across all 23,934 gene-level expression profiles. For each sample, the median Pearson correlation with all remaining samples was calculated as a summary measure of overall expression similarity. All 42 samples were retained in the primary analysis. GSM4556852, an AD sample, exhibited the lowest median correlation with the remaining samples and was therefore evaluated separately in an exclusion-based sensitivity analysis rather than being removed a priori.

### 4.5. Differential-Expression Analysis

Differential expression was evaluated using gene-wise linear models combined with empirical-Bayes variance moderation. Gene-wise residual variances were moderated toward a pooled prior variance using a common-degrees-of-freedom empirical-Bayes framework. This framework was implemented independently in Python and was methodologically analogous to the variance-moderation approach described in limma [29]; the R/limma package itself was not directly executed in the present analysis. Ordinary least-squares regression coefficients, standard errors, and associated statistics were independently cross-checked using statsmodels version 0.14.6. The calculations were implemented in Python version 3.13.5 (Python Software Foundation, Beaverton, OR, USA); the R/limma package itself was not directly executed. Ordinary least-squares regression coefficients, standard errors, and associated statistics were independently cross-checked using statsmodels version 0.14.6 to assess the consistency of the underlying linear-model estimates. 

The primary differential-expression model included diagnostic group, age, and sex as explanatory variables. Five pairwise contrasts were evaluated: DLB versus AD, DLB versus PDD, DLB versus neurologically unaffected controls, AD versus controls, and PDD versus controls. This contrast structure enabled assessment of both direct disease–disease differences and disease-associated expression changes relative to neurologically unaffected controls. For DLB-centered comparisons, positive log2 fold-change values indicated higher expression in DLB, whereas negative values indicated lower expression in DLB.

For each contrast, *p*-values were adjusted across all 23,934 tested genes using the Benjamini–Hochberg procedure [30]. A Benjamini–Hochberg false discovery rate (FDR) < 0.05 was considered the threshold for transcriptome-wide statistical significance. No absolute log2 fold-change threshold was required for the primary definition of differential expression. For descriptive purposes, an additional subset of FDR-supported genes with |log2FC| ≥ 0.5 was recorded. This effect-size criterion was used only for descriptive stratification and was not considered an additional statistical significance threshold.

### 4.6. Sensitivity Analyses

Several sensitivity analyses were conducted to evaluate the robustness of the principal differential-expression findings, with particular emphasis on the DLB-versus-PDD comparison. First, the analyses were repeated using a diagnosis-only model without adjustment for age and sex. Second, the primary age- and sex-adjusted model was repeated after exclusion of GSM4556852, the sample showing the lowest median correlation in the quality-control assessment.

Because PMI was unavailable for one PDD individual, additional analyses were performed in the 41 participants with complete PMI information. Within this identical complete-case subset, the age- and sex-adjusted model was first repeated without including PMI. PMI was then added as an additional covariate to assess whether the leading findings were robust to postmortem interval adjustment. An unmoderated ordinary linear-model analysis was also examined to assess the extent to which the main findings depended on empirical-Bayes variance moderation.

Two additional post hoc robustness analyses were performed. First, an expression-filtered analysis retained genes with mean log2 expression >5 across samples, reducing the tested set from 23,934 to 21,766 genes. Second, the analysis was restricted to the 23,479 gene symbols whose contributing PSRs mapped to a single transcript cluster. In both analyses, the same disease contrasts and multiple-testing procedures were applied. These analyses were regarded as sensitivity assessments rather than independent discovery analyses.

### 4.7. Definition of the Exploratory Cross-Disease Candidate Set

A stringent criterion was initially applied to identify DLB-associated disease-discrimination candidates, requiring FDR < 0.05 in both the DLB-versus-AD and DLB-versus-PDD comparisons together with a concordant direction of effect. Because no individual gene fulfilled these criteria, a separate post hoc exploratory analysis was subsequently performed to identify genes showing consistent, although weaker, evidence of DLB-associated differential expression across disease comparisons.

For inclusion in the exploratory set, genes were required to show nominal *p* < 0.05 in both the DLB-versus-AD and DLB-versus-PDD comparisons, concordant effect directions across these two contrasts, and FDR < 0.05 in the DLB-versus-control comparison. These criteria identified 79 genes. To further prioritize candidates showing more substantial disease–disease expression differences, an additional threshold of |log2FC| ≥ 0.25 was applied to both the DLB-versus-AD and DLB-versus-PDD contrasts, yielding a final set of 60 exploratory candidates.

This selection strategy was explicitly considered post hoc and hypothesis-generating. Accordingly, inclusion in the 60-gene set was not interpreted as evidence of transcriptome-wide statistical significance or as constituting a validated DLB-discriminatory molecular signature. Transcriptome-wide adjusted *p*-values from the primary analyses were therefore retained and reported independently of the exploratory selection criteria.

### 4.8. Integration with Published LBD Genetic and Multi-Omics Evidence

To assess overlap between the transcriptomic findings and published LBD genetic and multi-omics prioritization evidence, candidate genes were compared with genes prioritized by Zhu et al. [7]. The source study integrated genome-wide association results with multiple functional and molecular evidence layers and reported 85 prioritized LBD-associated genes.

The 85 reference genes were mapped to the 23,934 gene-symbol profiles retained in the GSE150696 discovery dataset. Exact gene-symbol matches were used whenever possible. Curated alias mappings were accepted only when supported by source annotation or established gene identifiers. Genes that could not be uniquely mapped to the discovery matrix were classified as unresolved rather than being interpreted as absent from the biological dataset.

Of the 85 source genes, 69 were mapped to the discovery expression universe, including 62 exact symbol matches and seven curated alias mappings; 16 remained unresolved. Overlap with the mapped LBD-prioritized gene set was evaluated across five transcriptomic gene sets: (i) the three genes FDR-supported in the primary age- and sex-adjusted DLB-versus-PDD model, (ii) the final 60-gene exploratory cross-disease candidate set, (iii) the broader 79-gene exploratory set obtained before application of the additional effect-size threshold, (iv) the FDR-supported gene set from the DLB-versus-control comparison, and (v) the FDR-supported gene set from the AD-versus-control comparison. Two-sided Fisher’s exact tests were used to assess the association between membership in each transcriptomic gene set and membership in the mapped LBD-prioritized gene set within the 23,934-gene discovery universe.

The individual evidence components assigned to overlapping genes in the Zhu et al. [7] prioritization framework—including GWAS significance, gene mapping, gene-based analyses, transcriptome-wide association studies, colocalization, summary-data-based Mendelian randomization, proteome-wide association studies, gene-set enrichment, drug–gene interaction, and gene-expression evidence—were examined separately. These categories were regarded as components of the source prioritization framework and were not interpreted as statistically independent replications.

### 4.9. Cell-Type-Resolved Transcriptomic Context from GSE178146

Cell-type-resolved transcriptomic evidence was evaluated using the published single-nucleus RNA-sequencing study corresponding to GSE178146 by Feleke et al. [9]. The source study analyzed anterior cingulate cortex tissue from 28 individuals, comprising seven neurologically unaffected controls and seven individuals each with Parkinson’s disease, PDD, and DLB. The published analysis characterized disease-associated transcriptional alterations across major neuronal and non-neuronal cell populations and provided a cellular framework for comparing molecular abnormalities among Lewy body diseases. In the present study, GSE178146 was used for biological and cell-type-resolved contextualization rather than as a formal independent gene-level replication cohort. Raw or donor-level single-nucleus counts were not reanalyzed, and nuclei were therefore not treated as independent experimental observations in the present statistical analysis.

This distinction was also maintained because GSE178146-derived expression information contributed to the expression-related evidence included in the multi-omics prioritization framework of Zhu et al. [7]. Consequently, the published GSE178146 findings and the expression component of the Zhu et al. [7] prioritization were not counted as two independent validation events.

### 4.10. Evidence Classification and Interpretation

To avoid combining findings with different statistical support, evidence was classified hierarchically. Genes reaching BH-FDR < 0.05 in a primary transcriptome-wide contrast were considered FDR-supported transcriptomic findings. Genes selected through the 60-gene post hoc procedure were classified as exploratory cross-disease candidates. Overlap with the Zhu et al. [7] gene-prioritization resource was recorded as exploratory overlap with external genetic prioritization and did not establish causal involvement, validation, or diagnostic utility.

Published GSE178146 findings were interpreted as cell-type-resolved external context and were not classified as independent gene-level replication unless a candidate-specific analysis had been independently reproduced from the underlying donor-level data. This hierarchical interpretation was used to prevent nominal, post hoc, and externally annotated evidence from being presented at the same level as transcriptome-wide FDR-supported findings.

### 4.11. Statistical Analysis

All statistical tests were two-sided unless otherwise specified. Transcriptome-wide differential-expression significance was defined as BH-FDR < 0.05. Nominal *p* < 0.05 was used only within analyses explicitly designated as exploratory.

For differential-expression analyses, effect estimates were reported as log2 fold changes together with raw and BH-adjusted *p*-values. Multiple-testing adjustment was conducted independently within each transcriptomic contrast. For descriptive overlap analyses, Fisher exact-test *p*-values and, where applicable, BH-adjusted values across the tested overlap configurations were retained separately. No machine-learning classifier, diagnostic prediction model, pathway-enrichment model, or molecular-signature validation procedure was used. Therefore, the results were interpreted as differential-expression and integrative candidate-prioritization findings rather than as evidence of clinical diagnostic performance. Statistical analyses were performed using Python version 3.13.5 (Python Software Foundation, Beaverton, OR, USA), with statsmodels version 0.14.6 used for independent ordinary least-squares verification of regression estimates. 

### 4.12. Statistical Power and Prospective Replication Sample-Size Analysis

To quantify the implications of the modest cohort size, a design-sensitivity analysis was performed for the two primary disease–disease contrasts. Using two-sided independent-group calculations at α = 0.05, the minimum standardized mean difference (Cohen’s d) detectable with 80% and 90% power was estimated for the observed group sizes (DLB *n* = 12 versus PDD *n* = 12; DLB *n* = 12 versus AD *n* = 9). Because Benjamini–Hochberg FDR control is data-adaptive and cannot be represented by a single fixed per-test α, these values were treated as conventional design-sensitivity estimates rather than exact transcriptome-wide FDR power calculations.

For prospective targeted replication of *SLC19A2*, *VAMP1*, and *COX5B*, approximate standardized effect sizes were derived from the primary-model log2 fold changes and their nominal moderated 95% confidence intervals. The standard error was approximated as (upper CI − lower CI)/(2 × 1.96), and Cohen’s d as |log2FC| × sqrt(1/n_DLB + 1/n_PDD)/SE. Required equal group sizes were first calculated from the observed discovery effects using a two-sided α = 0.0167 (0.05/3), corresponding to Bonferroni correction for the three prespecified genes. Because discovery-cohort effect sizes may be upwardly biased, a conservative planning scenario was also calculated after reducing each observed standardized effect by 50%. Calculations were performed for 80% and 90% power using statsmodels version 0.14.6 in Python version 3.13.5 and were intended for replication planning rather than as independent confirmation of the discovery findings.

## 5. Conclusions

The present study identified a limited set of candidate molecular differences among DLB, AD, and PDD rather than a robust transcriptome-wide disease-discriminatory signature. *SLC19A2*, *VAMP1*, and *COX5B* were FDR-supported in the primary age- and sex-adjusted DLB-versus-PDD model, although their statistical significance was dependent on model specification. *SYT11* was the sole exploratory overlap between the 60-gene transcriptomic candidate set and the published LBD-prioritized gene set; this overlap was not supported by significant enrichment and requires independent validation. Overall, the study did not detect broad robust disease–disease differences, a finding consistent with substantial molecular overlap but also compatible with limited statistical power. Larger independent, neuropathologically characterized, and cell-type-resolved cohorts are required to validate these observations. Conservative prospective calculations indicate that targeted replication of the three principal DLB-versus-PDD candidates would require approximately 20 participants per group for 80% power or 25 per group for 90% power. Future studies integrating single-cell multi-omics, proteogenomics, and interpretable machine-learning approaches may help resolve cell-type-specific disease mechanisms and improve molecular stratification across Lewy body- and Alzheimer-related dementias.

## Figures and Tables

**Table 1:** Summary of differential-expression findings in GSE150696.

Comparison	FDR < 0.05 Genes	Higher in First Group	Lower in First Group	FDR < 0.05 and |log2FC| ≥ 0.5
DLB vs. AD	0	0	0	0
DLB vs. PDD	3	0	3	3
DLB vs. CTRL	1806	307	1499	1304
AD vs. CTRL	612	105	507	473
PDD vs. CTRL	0	0	0	0

Abbreviations: AD, Alzheimer’s disease; CTRL, neurologically unaffected control; DLB, dementia with Lewy bodies; PDD, Parkinson’s disease dementia; FDR, false discovery rate; log2FC, log2 fold change.

**Table 2:** Principal DLB-associated transcriptomic candidates identified in the discovery analysis.

Gene	DLB vs. AD log2FC	DLB vs. AD BH-Adjusted *p*	DLB vs. PDD log2FC	DLB vs. PDD 95% CI	DLB vs. PDD BH-Adjusted *p*	DLB vs. CTRL log2FC	DLB vs. CTRL BH-Adjusted *p*
*SLC19A2*	−0.341	0.7674	−0.670	−0.912 to −0.427	0.02064	−0.530	0.01193
*VAMP1*	−0.218	0.9296	−1.299	−1.771 to −0.828	0.02064	−0.820	0.04158
*COX5B*	−0.296	0.8628	−0.869	−1.191 to −0.547	0.02064	−0.567	0.03871

Abbreviations: AD, Alzheimer’s disease; CTRL, neurologically unaffected control; DLB, dementia with Lewy bodies; PDD, Parkinson’s disease dementia; BH-adjusted *p*, Benjamini–Hochberg-adjusted *p*-value; CI, confidence interval; log2FC, log2 fold change. Negative log2FC values indicate lower expression in DLB relative to the comparison group. The reported 95% CIs for the DLB-versus-PDD contrast are nominal moderated confidence intervals and were not adjusted for multiple testing.

**Table 3:** Summary of evidence for the principal transcriptomic candidates and the exploratory *SYT11* overlap.

Gene	DLB vs. PDD	DLB vs. AD	DLB vs. CTRL	Exploratory 60-Gene Set	Published LBD-Prioritized Gene-Set Overlap	Interpretation
*SLC19A2*	FDR < 0.05	Nominal *p* < 0.05	FDR < 0.05	Included	No	Primary DLB-versus-PDD transcriptomic candidate; also included in the exploratory cross-disease set.
*VAMP1*	FDR < 0.05	*p* ≥ 0.05	FDR < 0.05	Not included	No	Primary DLB-versus-PDD transcriptomic candidate.
*COX5B*	FDR < 0.05	*p* ≥ 0.05	FDR < 0.05	Not included	No	Primary DLB-versus-PDD transcriptomic candidate.
*SYT11*	Nominal *p* < 0.05	Nominal *p* < 0.05	FDR < 0.05	Included	Yes	Sole exploratory overlap with the published LBD-prioritized gene set; hypothesis-generating.

Note: FDR-supported findings refer to the primary age- and sex-adjusted model. Nominal *p* < 0.05 does not indicate transcriptome-wide statistical significance. The published LBD-prioritized gene set refers to Zhu et al. [7]. GSE178146 was used only as cell-type-resolved contextual evidence and not as independent candidate-level replication. Abbreviations: AD, Alzheimer’s disease; CTRL, neurologically unaffected control; DLB, dementia with Lewy bodies; PDD, Parkinson’s disease dementia; FDR, false discovery rate; LBD, Lewy body dementia.

## Data Availability

The original contributions presented in this study are included in the article and Supplementary Materials. Further inquiries can be directed to the corresponding author.

## Supplementary Materials

The following supporting information can be downloaded at https://www.mdpi.com/article/10.3390/ijms27209173/s1.
